# Assessment of left atrial function using two-dimensional speckle tracking echocardiography in cryptogenic stroke patients

**DOI:** 10.1186/s43044-024-00563-6

**Published:** 2024-10-08

**Authors:** Amr Setouhi, Tarek Mohamed Abdelrahman, Ahmed Mohamed Ali, Mohamed Abdelkadir Abdelwahab

**Affiliations:** Department of Cardiology, Faculty of Medicine, Minia University, Minya, Egypt

**Keywords:** Cryptogenic stroke, Atrial fibrillation, Left atrial, Left atrial reservoir strain rate, Speckle tracking echocardiographic, Left atrial volume index

## Abstract

**Background:**

Cryptogenic cerebrovascular stroke can be defined as an ischemic stroke that lacks a clear cause, even after a thorough evaluation. It should be distinguished from the embolic stroke of undetermined source (ESUS), a subgroup that includes cardio-embolic sources. This study aims to assess left atrial function through two-dimensional speckle tracking echocardiography (2D-STE) to determine its potential association with cryptogenic stroke and its predictive value for subclinical atrial fibrillation (AF). Our prospective cohort study involved 62 patients with unexplained cerebrovascular stroke or TIA, regardless of gender. Following TEE assessments, 22 patients were excluded due to identified sources of cardio-embolism. The remaining 40 participants were clustered into Group I. Group II, consisted of 40 healthy individuals without significant medical history, served as a control group. Both groups underwent two-dimensional trans-thoracic echocardiography and speckle tracking echocardiography.

**Results:**

LA dysfunction parameters exhibited significant differences between Group I and Group II. LV diastolic dysfunction, LAVI, LAEF, and LASr were notably affected in Group I. At the same time, LA diameter in the parasternal long-axis view (PLAX) displayed a significant difference with a p value of 0.001. Within Group I, 14 patients experienced AF episodes (Group Ia, 35%); while, the remaining 26 were categorized as Group Ib (65%). LV diastolic dysfunction displayed a p value < 0.011; while, LAVI, LAEF, and LASr exhibited considerable differences with p values < 0.0001. However, the LA diameter showed no significant variation between the two groups. LASr emerged as the most sensitive and specific parameter for predicting AF, with a cutoff point of ≤ 24.5% and a p value < 0.0001. LAEF showed a cutoff point of ≤ 40.5% and a p value of 0.011. Meanwhile, LAVI demonstrated the lowest sensitivity and specificity, with a mean cutoff point of ≥ 38.5 ml/m^2^ and a p value of 0.003.

**Conclusions:**

2D-STE is crucial for assessing LA dysfunction as a potential cryptogenic stroke cause after TEE and ruling out cardio-embolism sources. LASr serves as a key LA cardiopathy indicator, even preceding AF. LASr independently poses an AF risk. While LAEF and LAVI are significant LA dysfunction parameters and AF predictors, they exhibit lower sensitivity and specificity than LASr.

## Background

Ischemic cerebrovascular stroke is a fatal worldwide condition; determining its etiology is important for therapeutic purposes or preventing further episodes. Although there are advanced diagnostic tools, some cerebrovascular stroke etiologies could not be identified; this is termed cryptogenic stroke (CS). Researchers specified the cryptogenic stroke embolic subtype as an Embolic Stroke of Undetermined Source (ESUS) [1]. Cardiac evaluation is important in patients with ESUS, as previous studies have shown that cardio-embolic strokes constitute approximately one-third of ischemic strokes [2]**.**

Various mechanisms may drive CS, but the most significant clinical predictors for CS are often overlooked: atrial fibrillation (AF) and subclinical atherosclerotic disease. These factors are closely linked to left atrial mechanical dysfunction, called left atrial cardiopathy [3]. The two-dimensional speckle tracking echocardiographic imaging (2D-STE) of the left atrium assesses sensitive myocardial deformation (LA strain) involving LA function analysis. Impaired LA strain is observed in patients with one or more atherosclerotic risk factors despite normal LA size or dimension [4]. So, 2D-STE of LA may be of extreme value in patients with CS.

Trans-esophageal echocardiography (TEE) is also an essential tool in the workup of cryptogenic stroke. Numerous studies have assessed the effectiveness of transesophageal echocardiography (TEE) and non-invasive transthoracic echocardiography (TTE) in patients. TEE has excellent effectiveness and sensitivity for detecting LA appendage thrombus, PFO, or aortic atheroma [5]. Some researchers may consider the TEE as a routine investigation in younger patients (< 55 years old) with ischemic stroke [6].

So, explaining the pathogenesis of cryptogenic stroke is more than an academic issue, as each specific stroke subtype often guides different secondary stroke prevention or therapeutic strategies [3].

The study aims to evaluate LA function in cryptogenic stroke patients by employing two-dimensional speckle tracking echocardiography and investigate the parameters of left atrial dysfunction as a possible etiology for cryptogenic stroke.

## Methods

 This prospective cohort study enrolled 62 patients who were presented with cerebrovascular stroke or TIA and were referred to the cardiology department for cardiac evaluation. Another 40 participants with no previous medical history of implication were included as a control group for comparison of left atrial function to those patients with cryptogenic stroke.

So, participants were divided into two groups after conducting TEE for all participants and exclusion of possible etiologies of cardio-embolism: Group I (40 cryptogenic stroke patients) and Group II, which included healthy participants as the control group. Participants of Group I were divided into Group Ia (AF recorded group) and Group Ib (non-AF registered group). Our study was approved by the Local Institutional Human Research Ethics Committee, and participants provided written informed consent.

This study included female and male patients with confirmed cerebrovascular ischemic stroke, diagnosed through brain imaging (utilizing brain CT or MRI to rule out primary intracranial hemorrhage), and individuals who experienced transient ischemic attacks.

Exclusion criteria for cardio-embolism/stroke risk include patients with persistent or paroxysmal atrial fibrillation (all participants with paroxysmal atrial fibrillation less than 30 s were excluded), aortic or mitral valvular metallic prostheses, moderate or severe valvular lesions, a history of CAD, LVEF < 50%, intra-cardiac mural thrombi or shunts, significant carotid artery stenosis (> 50% by sonography), uncontrolled hypertension, known aortic aneurysms, metabolic or electrolyte disorders, thyroid dysfunction (a risk factor for atrial fibrillation), and a history of recognized stroke causes like Polycythemia rubra vera or Anti-phospholipid syndrome. These criteria are vital for patient selection in medical interventions or studies, ensuring safety and minimizing embolic risk.

All patients were subjected to the following screening steps:

(i) Full history tracking:
  - Risk factors such as gender, age, smoking, diabetes mellitus, hypertension, dyslipidemia, and positive family history of the ischemic event, either cerebral vascular or cardiac event.
  - Drug history, including medications and doses.
  - Previous and existing cardiac problems like ischemic heart diseases and heart failure.
  - Other medical problems.
(ii) General and local cardiac examination:
  It involves arterial blood pressure measurement, body weight, height, pulse, body mass index, and body surface area.
(iii) Resting 12-leads surface ECG: to exclude arrhythmia, atrial fibrillation, paroxysmal or persistent CAD, or cardiac chambers dysfunction, and also for detection of left atrial cardiopathy markers as P wave duration, PTFV1.
(iv) Laboratory: the routine laboratory investigations in patients with cerebrovascular stroke as complete blood count, renal and hepatic profiles, coagulation profile, and total Lipid profile parameters, including serum triglycerides, serum cholesterol, high-density lipoprotein cholesterol, low-density lipoprotein cholesterol, and non-HDL cholesterol.

Echocardiography was performed using The ACUSON SC2000 PRIME ultrasound system, Siemens, Germany. An echocardiographic study was done using 2D, M-mode, and Doppler and tissue Doppler techniques. All study participants were examined while in the left lateral recumbent position and instructed to refrain from deep inhalation or performing the Valsalva maneuver. Systolic and diastolic functions of LV were assessed, LA anteroposterior diameter was measured in PLAX view, LA maximal and minimal volumes in biplane (apical 4 and apical 2 chambers) view, LA volume index (LAVI) which was considered by (LA maximal volume / BSA). Also, the LA emptying volume was evaluated by assessing the LA maximum and LA minimum volume difference. LA ejection fraction (LAEF) was estimated by the LA emptying/maximum volume.

LV diastolic function was assessed by mitral inflow pattern in apical 4 chambers view and calculating E/A ratio. 'E' wave represents the early ventricular diastole, and 'A' wave represents the atrial contraction and septal tissue Doppler velocities to estimate e`/a,` e` represents early diastolic annular velocity, a' represents the annular late diastolic velocity, following the European Association of Cardiovascular Imaging and the American Society of Echocardiography recommendations, updated in 2016.

For TEE evaluation, an ultrasound system using (Siemens Acuson SC2000) using a "Z6Ms" transducer or (Philips iE33) using an "xMATRIX TEE X 7-2t" transducer was used. All of the patients were in fasting condition for a minimum of 4 h before the procedure. Oxygen saturation and blood pressure were evaluated. Local pharyngeal anesthesia with 2% lidocaine spray was used along with low-dose intravenous midazolam.

Standardized transesophageal echocardiography (TEE) images were acquired using mid-esophageal views, including the 4 chamber, mitral commissural, 2 chamber, long axis, ascending aorta long axis, aortic valve short axis, bicaval, and right ventricular inflow–outflow views. Furthermore, a multi-planar assessment of the left atrial appendage (LAA) was implemented. In cases where the inter-atrial shunt was not observed via color flow Doppler in the bicaval view, agitated intravenous saline was managed for further assessment. Furthermore, additional standard images of the descending aorta and aortic arch in both short and long axes were captured to complete the evaluation. LA and LAA in multiple angles were evaluated for spontaneous echocardiographic contrast (SEC) and/or thrombus formation. Thrombus was identified as echodensity with distinct borders, observed in various views, and autonomous from an endocardium. SEC was defined with the echo-dense smoke-like motion.

A saline contrast study was administered through normal respiration and the Valsalva maneuver to assess the inter-atrial septum accurately. The PFO presence was established based on micro-bubbles direct visualization passing through the atrial septum to LA by recording three consecutive cardiac cycles after complete opacification of the right atrium or detecting color Doppler flow through the atrial septum. The use of TEE in our study searches for objective evidence for embolic stroke as a routine workup in cryptogenic stroke to exclude cardio-embolic sources such as PFO, LAA appendage thrombi, or even SEC or significant aortic plaques.

High-risk PFO for paradoxical embolism is defined as Large PFO size (septum primum maximum separation from the secundum throughout the Valsalva maneuver) ≥ 2 mm on TEE, presence of inter-atrial septal aneurysm (dilated segment protrusion of the septum at least 15 mm beyond the atrial septum level surface) or hypermobility. Significant aortic arch/ ascending aorta plaques were defined as ≥ 4 mm in thickness.

Speckle tracking study for LA was performed on all members of the study population. From the apical 4 chamber and 2 chamber views 202 with a stable ECG recording, the left atrium will be visualized as clearly as possible, permitting reliable delineation of myocardial tissue and extra-cardiac structures. The non-foreshortened tracing was started at the mitral annulus endocardial border, extrapolating across the pulmonary veins and/or LA appendage orifices by employing the apical four chamber. Most studies recommend biplane apical 2 and apical 4 chambers views in echocardiographic assessment of LA by 2D-STE; however, using a single apical view (apical 4 chambers view) may be accepted and provides the LA strain normal reference values throughout the reservoir, contraction phases, and conduit. Cine-loops of the left atrium (LA) from well-optimized LA-focused images ensure the maximum LA length and base inclusion in each view.

A frame rate of 40–90 frames/three consecutive cardiac cycles was used. LA Cine-loops were captured from well-optimized LA-focused images to maximize the inclusion of the length and base of LA. The used settings were selected to merge temporal resolution with suitable spatial definition and improve the frame-to-frame tracking technique feasibility. Images are recorded and analyzed offline using the speckle tracking software.

The aim of using 2D Speckle tracking echocardiography in our study is to assess left atrial mechanical dysfunction when TEE could not explain the source of cardio-embolism and as an important diagnostic tool for early prediction of subclinical AF 205. The left atrial ejection fraction (LAEF) and left atrial reservoir strain rate (LASr) were used to evaluate left atrial function.

Bilateral carotid artery ultrasonography was performed on all participants of the study. All patients with significant carotid artery atheromatous plaques with more than 50% stenosis were excluded. Plaque was characterized as a localized structure protruding into the arterial lumen, displaying a thickness exceeding 2 mm, evaluated from the interface between the intima and lumen to the interface between the media and adventitia. Non-obstructive carotid atherosclerosis (NOCA) is less than 50% of carotid artery atherosclerosis.

### ECG rhythm monitoring during hospitalization

All participants were monitored for ECG rhythm abnormality for 48 h during hospitalization in the stroke unit. Episodes of atrial fibrillation more than 30 s during the 48 h were recorded.

## Results

**Fig. 1:**
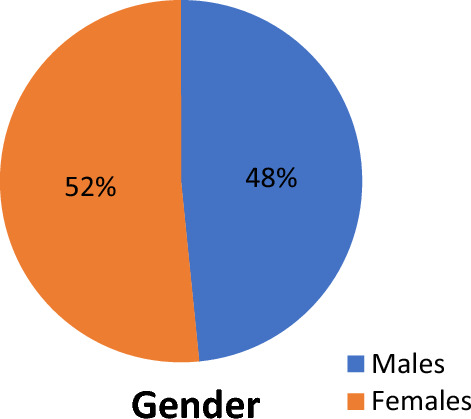
Patient's gender

Our prospective cohort study was performed on 62 patients of both sexes proved to have an ischemic stroke of undefined cause, 'cryptogenic stroke,' or TIA, admitted to the Neuro-psychiatric department. 30 patients (48.4%) were males and 32 (51.6%) were females, as shown in Fig. 1.

**Fig. 2:**
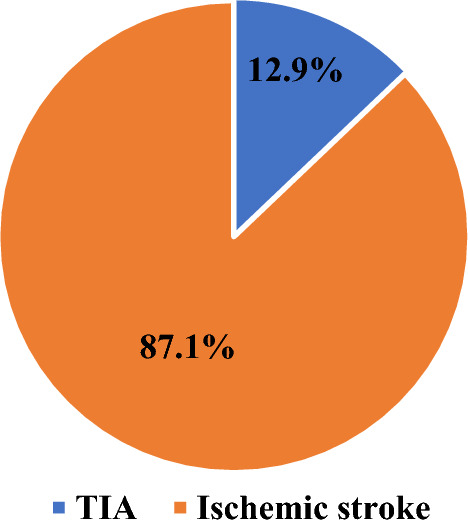
Patient's presentation

Cerebro-vascular infarctions were presented 54 patients (87%); while, 8 (13%) patients were presented with the transient ischemic attack (TIA), as shown in Fig. 2. Table 1 provides an overview of the study participants' demographic data, comorbidities, and risk factors.

**Table 1:** Demographic data, comorbidities, and risk factors

		Cases N = 62
Gender		
Males		30 (48.4%)
Females		32 (51.6%)
Age	Mean ± SD	39.8 ± 9.8
Risk factors		
DM		18 (29.0%)
Hypertension		24 (38.7%)
Further comorbidities:		
Collagen disease		14 (22.5%)
Chronic chest disease		2 (3%)
CKD		2 (3%)
Smoking		18 (29.0%)
Family history		14 (22.6%)
BMI (Kg/ m^2^)	Mean ± SD	28.2 ± 5.4
BSA (m^2^)	Mean ± SD	1.9 ± 0.2
Lipid profile		
Cholesterol (mg/dl)	Mean ± SD	219.6 ± 45.7
TGs (mg/dl)		171.0 ± 34.2
LDL cholesterol (mg/dl)		139.6 ± 24.8
HDL cholesterol (mg/dl)		54.0 ± 15.9
Non-HDL cholesterol (mg/dl)		145.1 ± 28.2

32 (52%) patients were found to have non-obstructive carotid stenosis (<50% stenosis of any of the carotid arteries); while, 30 patients (48%) had normal carotid ultrasonography. Patients with significant carotid stenosis (plaques with more than 50% stenosis) were excluded. Trans-esophageal echocardiography was performed on all participants, assessing LAA, aortic arch, and inter-atrial septum, seeking an embolic source to explain the etiology. 22 participants were excluded due to the presence of a possible source or objective evidence that could explain the etiology of cerebrovascular stroke, as shown in Table 2. 8 participants were found to have patent foramen ovale (PFO), 4 of them were classified as low-risk PFO, and 4 were classified as high-risk PFO for paradoxical embolism according to the definition described before.

**Table 2:** Possible etiologies of CS detected by TEE

	N = 22
LAA thrombus	10
PFO	8
Significant aortic atheroma	4

The parameters of left atrial cardiopathy (LA diameter, LA volume index, LV diastolic dysfunction, LA ejection fraction, and LA strain rate during reservoir phase by 2D speckle tracking echocardiography) were compared in participants of cryptogenic stroke after exclusion of participants who had a possible source of cardio-embolism in TEE (n = 40, Group I). They were compared to another 40 healthy participants with no history of chronic illness (Group II, control group), as shown in Table 3.

**Table 3:** Comparison between LA cardiopathy parameters in Group I (CS) and Group II (control group)

	Group I (Cryptogenic stroke) N = 40	Group II (Control) N = 40	p value
Age			
Mean ± SD	41.70 ± 7.79	39.35 ± 6.56	0.001*
Gender (%)			
Male	50	50	
Female	50	50	
LV diastolic dysfunction			
Normal	12 (30.0%)	30 (75.0%)	
DD grade I	20 (50.0%)	8 (20.0%)	< 0.0001*
DD grade II	8 (20.0%)	2 (5.0%)	
LA diameter (cm)	3.8 ± 0.3	3.6 ± 0.2	< 0.001*
LAVI (ml/m^2^)	38.8 ± 5.1	29.8 ± 3.7	< 0.0001*
LAEF (%)	44.2 ± 6.5	54.4 ± 5.8	< 0.0001*
LASr (%)	26.1 ± 6.1	34.6 ± 4.8	< 0.0001*

^*^Significant level is p-value < 0.05

The patients of cryptogenic stroke (group I) were monitored by ECG during hospitalization for 48 h to detect episodes of atrial fibrillation over 30 s. This group was further divided into two groups according to the recording of episodes of AF; episodes of AF were seen in 14 patients (35%, Group Ia) and not recorded in 26 patients (65%, Group Ib), as shown in Table 4.

**Table 4:** Classification of patients according to AF recording by ECG monitoring during hospitalization in Group I

AF	
AF recorded (group Ia)	14 (35%)
Non-AF recorded (Group Ib)	26 (65%)

Parameters of atrial cardiopathy were compared in both Group Ib (non-AF recorded group) and Group II (control group), as shown in Table 5.

**Table 5:** Comparison between LA cardiopathy parameters in Group II and Ib

	Group II N = 40	Group Ib N = 26	*P*-value
	Mean ± SD	Mean ± SD	
LA diameter (cm)	3.6 ± 0.2	3.8 ± 0.2	0.053
LA volume index (ml/m^2^)	29.8 ± 3.7	36.9 ± 4.9	< 0.0001*
LASr (%)	34.6 ± 4.8	28.9 ± 5.3	< 0.0001*
LAEF (%)	54.4 ± 5.8	46.1 ± 7.2	< 0.0001*
LV diastolic function	N (%)	N (%)	
Normal	30 (75.0%)	10 (38.5%)	
DD grade I	8 (20.0%)	14 (53.8%)	0.011*
DD grade II	2 (5.0%)	2 (7.7%)	

^*^Significant level is p-value < 0.05

**Fig. 3:**
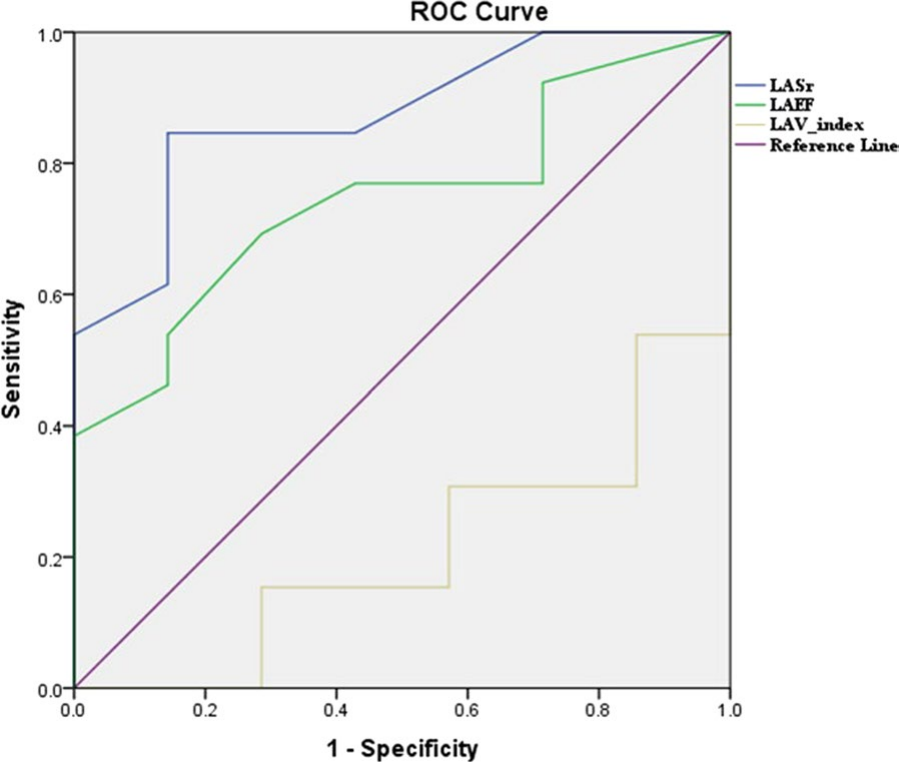
ROC curve for sensitivity and specificity of predictors of AF

Parameters of left atrial cardiopathy in Group Ia were compared by ROC curve to determine their specificity and sensitivity for AF prediction, as shown in Fig. 3 and Table 6.

**Table 6:** ROC curve of LASr, LAEF, and LAVI for prediction of AF

	LASr	LAEF	LAVI
AUC (Area under the curve)	0.874	0.714	0.209
P value	< 0.0001*	0.011*	0.003*
The cutoff point for AF prediction	≤ 24.5%	≤ 40.5%	≥ 38.5 ml/m2

^*^Significant level is p-value < 0.05

The LASr is the utmost sensitive and definite parameter of LA cardiopathy in predicting AF with a cutoff point ≤ 24.5% and p value < 0.0001, then LAEF with a cutoff point ≤ 40.5% and p value = 0.011. The LAVI is the least sensitive and specific parameter with a mean cutoff point of ≥ 38.5 ml/m^2^ and p value = 0.003, as shown in Table 6.

Tables 7, 8, and 9 present sensitivity and specificity values and their 95% confidence intervals for different predictive parameters in the context of atrial fibrillation (AF). These tables assess LASr, LAEF, and LAVI, providing valuable insights into their diagnostic accuracy. Table 10 offers a comparative analysis between cases with AF and those without AF, highlighting differences in left atrial cardiopathy parameters, including LA diameter, LAVI, LAEF, and LASr, as well as the distribution of diastolic dysfunction grades. Significant findings are indicated by asterisks (p value < 0.05).

**Table 7:** Sensitivity and specificity of LASr in the prediction of AF

Statistic for LASr	Value (%)	95% CI
Sensitivity	84.62	65.13–95.64%
Specificity	85.71	57.19–98.22%
Positive Predictive Value	91.67	75.11–97.57%
Negative Predictive Value	75.00	54.29–88.34%
Accuracy	85.00	70.16–94.29%

**Table 8:** Sensitivity and specificity of LAEF in the prediction of AF

Statistic for LAEF	Value (%)	95% CI
Sensitivity	76.92	56.35–91.03%
Specificity	57.14	28.86–82.34%
Positive Predictive Value	76.92	63.73–86.35%
Negative Predictive Value	57.14	36.63–75.46%
Accuracy	70.00	53.47–83.44%

**Table 9:** Sensitivity and specificity of LAVI in the prediction of AF

Statistics for LAVI	Value (%)	95% CI
Sensitivity	53.85	33.37–73.41%
Specificity	14.29	1.78–42.81%
Positive Predictive Value	53.85	43.51–63.86%
Negative Predictive Value	14.29	4.15–39.10%
Accuracy	40.00	24.86–56.67%

**Table 10:** Comparison between parameters of left atrial cardiopathy in AF recorded group and non-AF recorded group

Parameters		Cases with AF (group Ia) N = 14	Cases without AF (group Ib) N = 26	p value
LA diameter (cm)	Mean ± SD	3.9 ± 0.2	3.7 ± 0.2	0.12
LAVI (ml/m^2^)	Mean ± SD	42.4 ± 3.0	36.8 ± 4.9	< 0.0001*
LAEF (%)	Mean ± SD	40.6 ± 2.4	46.0 ± 7.2	0.012*
LASr (%)	Mean ± SD	21.2 ± 3.8	28.8 ± 5.3	< 0.0001*
DD				
Normal		2 (14.2%)	10 (38.5%)	
Grade I	N %	6 (42.9%)	14 (53.9%)	0.158
Grade II		6 (42.9%)	2 (7.6%)	

^*^Significant level is p-value < 0.05

Table 10 provides a comparative analysis between two groups: Cases with atrial fibrillation (AF) (Group Ia, N = 14) and cases without AF (Group Ib, N = 26). The table evaluates various parameters related to left atrial cardiopathy, including left atrial (LA) diameter, left atrial volume index (LAVI), left atrial emptying fraction (LAEF), and left atrial strain (LASr). Additionally, it categorizes diastolic dysfunction (DD) into normal, Grade I, and Grade II. Notably, significant differences were observed in LAVI, LAEF, and LASr between the two groups, as indicated by p values < 0.05, highlighting the relevance of these parameters in assessing left atrial cardiopathy concerning AF.

## Discussion

Cryptogenic cerebrovascular stroke is an ischemic stroke without a defined cause despite a complete workup. It differs from Embolic Stroke of Undetermined Source (ESUS), a subgroup of cryptogenic stroke described as embolic stroke without definite cardio-embolic origin [7]. This prospective cohort study aimed to assess the left atrial mechanical dysfunction by two-dimensional Doppler echocardiography and speckle tracking echocardiography as a possible reason for cryptogenic cerebral vascular stroke. The left atrial mechanics and parameters of left atrial cardiopathy were investigated as a possible causative mechanism for cryptogenic stroke after performing a TEE study to complete the workup for cryptogenic stroke and exclude potential sources for cardio-embolism.

Our study included 62 participants of both genders with an unexplained cerebral vascular stroke or transient ischemic attack (TIA) for cardiological workup. The average age of included patients was 39.8 ± 9.8 years, indicating that cryptogenic stroke is more common in younger ages after excluding documented causes for cerebral stroke, such as significant cerebral vessel atherosclerosis, LV cardiomyopathy, or atrial fibrillation, as all these factors are associated with older age than younger. 22 participants were excluded after conducting the TEE study (10 with LAA thrombus, 8 with PFO, and 4 with significant aortic atheroma, as shown in Table 2**.** Regarding risk factors of atherosclerosis, our study included 24 hypertensive patients (38.7%), 18 diabetic patients (29%), and 18 smoker patients (29%). The main result of this study is that the parameters of LA cardiopathy, such as LAVI, LAEF, and LASr, were significantly impaired in patients with cryptogenic stroke compared to controls.

Furthermore, all these parameters were significantly affected in the subgroup' non-AF recorded patients' compared with the healthy control group. Moreover, it was found that all three parameters were affected to a higher extent in the AF group compared to the non-AF Group. This means LA cardiopathy may contribute to cryptogenic stroke even without atrial fibrillation.

Our results revealed that 32 patients in Group I had non-obstructive carotid atherosclerosis (NOCA) (an atheromatous plaque) with less than 50% stenosis of carotid arteries. This agrees with Buon et al. [8], who conducted a study of 44 patients with carotid CS and found NOCA in 22 (50%). Furthermore, Jaffre et al. [9] studied over 192 patients aged 18–54 to evaluate the prevalence of NOCA and PFO in cryptogenic stroke. They found that NOCA is common in young adults with CS and that NOCA detection is essential for investigating stroke potential in young adults. Similarly, Bulwa and Gupta [10] confirmed that non-stenotic plaques of carotid arteries are causative in strokes of undetermined etiology.

Tandon et al. [11] studied atrial fibrosis by late-gadolinium-enhancement MRI in patients with ESUS and with atrial fibrillation, and they found that ESUS patients experienced atrial fibrosis similar to the AF group, with more fibrosis compared with the control group indicating that fibrosis is in the contributory path of cardio-embolic stroke autonomously of AF. Also, in the same context, Camen and Schnabel [12] support the concept that left atrial cardiopathy has its clinical significance and recommend further trials to explain if LA cardiopathy could lead to thrombus formation even without experiencing AF.

By using the ROC curve to study the sensitivity and specificity of LA cardiopathy parameters in the prediction of AF, LASr is the most sensitive and specific parameter for the prediction of AF with a cutoff point < 24.5% with a sensitivity of 85% and specificity 86%, then LAEF with cutoff point < 40.5%. LAVI is the least sensitive and specific parameter for predicting AF with a mean cutoff point of > 38.5 ml/m^2^. Similar results were obtained from LE Sade et al. [13]**,** who studied the left atrial mechanics responsible for LA remodeling in CS and ESUS and found that LASr is more sensitive and specific in the prediction of AF with 90% sensitivity, 90% specificity and p value < 0.0001 in CS. In ESUS, the study found that LASr shows 86% sensitivity and 92% specificity with a p value < 0.0001 in the prediction of AF.

Ble et al. [14] confirmed the role of evaluation of left atrial function and strain by STE as an extremely valuable modality for assessing left atrial disease and seeking silent AF in patients with CS. Many studies concluded that the functional remodeling evaluated by reducing myocardial deformation is more adept at detecting subclinical atrial dysfunction than identifying atrial enlargement [15–17]. In agreement with our results, the I-LASER study [18] published in the AHA journal in 2021 also found that patients with improved LA reservoir function may experience fewer chances of cardio-embolic stroke despite the risk factors, demographics, and AF existence. The results indicated that measuring the LA reservoir provides a reliable method for accurate LA dysfunction capture.

It has been demonstrated in many other studies that impaired LA reservoir function in acute ischemic stroke patients evaluated using 2D-STE will grant an incremental predictive AF value [19]. Sonaglioni et al. [20] provided strong evidence that reduced left atrial strain during left ventricular systole, glomerular filtration rate, and the Rankin Scale at admission independently forecast all-cause mortality and rehospitalization due to cerebrovascular events in acute ischemic stroke individuals.

In retrospective cohort research, Deferm et al. [21] recently demonstrated that left atrial strain during left ventricular systole was notably reduced in cryptogenic stroke patients who subsequently developed atrial fibrillation (AF) compared to those without AF. The results indicate that the LA strain can be used as a surrogate marker for occult AF detection in cryptogenic stroke patients during LV systole. Similar results were reported by Olsen et al. [22]**,** who confirmed that LA strain drop throughout LV systole was linked with occult AF despite the LV longitudinal systolic strain and volume in cryptogenic stroke patients, as detected by insertable cardiac monitors**.** The LA diameter by 2D TTE was assessed by measurement of A-P diameter in PLAX view in cm as a parameter for atrial cardiopathy. LA diameter is significantly increased in Group I (Cryptogenic stroke group) compared to Group II (p value < 0.001). The I-LASER study [18], a study over 151 patients aimed to investigate the LA structure and its role in stroke etiology, demonstrated a significant association between larger LA volumes and cardio-embolic stroke.

Regarding LA volume as a parameter for left atrial cardiopathy and a risk factor for cardio-embolic stroke, Jordan et al. [23] conducted a study over 1020 patients and found that there is no significant difference in LAVI in patients with ESUS versus non-cardio-embolic stroke with p value = 0.61; while, there is significant value between ESUS versus cardio-embolic stroke with p value = 0.001. Similar conclusions were reported by Kamel et al. [24], who conducted a study of over 1293 patients in the Cornell Acute Stroke Academic Registry (CAESAR), whose results confirmed that the LAVI variation between cardio-embolic stroke and ESUS patients was remarkably greater than the variation observed between non-cardio-embolic stroke cases and ESUS. They also demonstrated that patients with high LAVI often develop AF and are classified as embolic stroke. While M Acampa et al. [25] classified their 226 patients into mild ESUS (NIHSS ≤ 5) and severe ESUS (NIHSS > 5). LAVI was significantly increased in severe ESUS compared to mild ESUS with p value = 0.04. NIHSS National Institutes of Health Stroke Scale is a clinical risk score for risk stratification of stroke severity and prediction of clinical outcome depending on the assessment of multiple clinical neurological functions as conscious level, motor, sensory responses, facial palsy, ataxia and dysarthria [26].

Our results denoted that LV diastolic dysfunction is not a strong parameter for predicting atrial fibrillation compared to the other studied parameters (p value = 0.158). SURPRISE echo sub-study results agree well with Olsen et al. [22], who demonstrated that LV diastolic dysfunction classical grading by E/A ratio is less efficient compared to other echocardiographic parameters for AC for the prediction of AF. Moreover, in an I-LASER study published in the AHA journal in 2021, no other significant associations were found between LV diastolic function and stroke of unknown cause [18].

On the other hand, Kim et al. [27] studied the effect of LV diastolic dysfunction on LA remodeling and risk of stroke and paroxysmal AF by echocardiographic and computed tomographic evaluation of the heart. They found that using the E/E` ratio for estimating LV filling pressure is linked with the LA structural remodeling and LAA emptying flow velocity, and not linked to a high risk of stroke /TIA in paroxysmal AF patients. Also, Park et al. [28] documented the importance of LV diastolic dysfunction assessment in patients after ischemic stroke, which was associated with poor outcomes regarding further vascular events or functional recovery.

## Conclusions

Cryptogenic stroke diagnostic workup is mandatory to reach a specific etiology that will differ in primary management and secondary prevention of further thrombo-embolism. Moreover, our study confirmed the important role of transesophageal echocardiographic study for detecting possible etiology that could explain cardio-embolism, such as LAA thrombi, large PFO, or significant aortic arches atheroma.

Furthermore, the study's results revealed that evaluating LA strain through the reservoir phase is a strong parameter for LA cardiopathy even before AF. LA strain during the reservoir phase is also an independent risk factor for AF. LA ejection fraction and LAVI are essential factors related to LA dysfunction and AF prediction with less sensitivity and specificity than LASr.

### Limitations and recommendations

The sample size of patients was relatively small. In addition, relatively short ECG rhythm monitoring during hospitalization may not be enough to detect all subclinical or paroxysmal AF.

Further studies with more patients and prolonged ECG monitoring for detecting subclinical AF are required. Longer follow-up of patients documented in this study to have left atrial cardiopathy to know if they will develop AF or not. Follow-up of Group I patients in this study after tight control of risk factors and lifestyle modification to detect the improvement or deterioration of left atrial cardiopathy. Based on the results of this trial, speckle tracking echocardiography with measurements of parameters of left atrial cardiopathy will be done for high-risk patients for stroke.

## Data Availability

The dataset used during the current study is available from the corresponding author upon reasonable request.

## Abbreviations

2D-STE: Two-dimensional speckle tracking echocardiography
AF: Atrial fibrillation
CAESAR: Cornell Acute Stroke Academic Registry
ESUS: Embolic stroke of undetermined source
LA: Left atrial
LAA: Left atrial appendage
LAEF: Left atrial ejection fraction
LASr: Left atrial reservoir strain rate
LAVI: Left atrial volume index
NOCA: Non-obstructive carotid atherosclerosis
PLAX: Parasternal long-axis view
TEE: Trans-esophageal echocardiography
